# Moving Beyond Lewis: Employment and Wage Trends in China’s High- and Low-Skilled Industries and the Emergence of an Era of Polarization: Presidential Address for the 2020 Association for Comparative Economic Studies Meetings

**DOI:** 10.1057/s41294-020-00137-w

**Published:** 2020-10-21

**Authors:** Scott Rozelle, Yiran Xia, Dimitris Friesen, Bronson Vanderjack, Nourya Cohen

**Affiliations:** 1 Stanford University, Stanford, USA; 2 School of Business, Wenzhou University, Wenzhou, China; 3 Rural Education Action Program, Freeman Spogli Institute, Stanford University, Stanford, USA

**Keywords:** Employment, Wages, Trends, Divergence, High-skilled, Low-skilled, J21, J31, O53, J24

## Abstract

One of the defining features of China’s economy over the two decades between 1995 and 2015 was the persistent rise of wages for workers and professionals in nearly every segment of the economy—with wage rates for labor-intensive jobs in manufacturing, construction, and the informal service sector rising the fastest. Recently, however, the economic environment in China has begun to change, including changes in both employment and wages. We identify recent employment/wage trends throughout China’s economy and postulate the sources of these trends as well as possible future consequences if they continue. We use official, nationally aggregated data to examine employment and wages in multiple sectors and industries. Our findings indicate that China may have entered a new phase of economic development in the mid-2010s. According to the data, in recent years, wage growth has begun to polarize: Rising for professionals employed in formal skill-intensive industries; and falling for workers in the informal labor-intensive service sector. We attribute this increase in skill-intensive wages to an increase in demand for skill-intensive employment, due to the emergence of a large middle class in China, for whom the demand for high technology, finance, banking, health, and higher education industries is increasing while, at least in the recent short term, the supply of experienced, high-skilled professionals has not kept up. The employment/wage trend in the informal (low-wage) service sector, however, is following a different pattern. While there is a rising demand for services in China’s economy, the growth, due to a number of factors (e.g., large shares of GDP targeted by policymakers to investment; high rates of savings by consumers), is relatively slow. In contrast, due to a number of economic forces, including globalization and automation, the supply of labor into the service sector of the informal economy is being fueled by the flow of labor out of manufacturing and construction (two industries that that have experienced employment declines since 2013). These supply and demand trends, in turn, are leading to the fall in the growth rate of wages in the informal service sector. We conclude by discussing the possible longer-term consequences of these emerging polarization trends based on an examination of recent experience with wage polarization occurring in both middle- and high-income countries, as well as its consequences. We also present policy recommendations for greater investment in education and human capital, as well as for the development of a more comprehensive set of social safety nets for different segments of China’s population.

## Introduction

Low wages were an important factor in China’s rapid economic growth and development, especially during the first decades after reform in the 1980s and 1990s. After the initial economic reforms, China opened itself to the outside world, triggering the initial rise of foreign direct investment (FDI). Along with a series of other policy initiatives, China launched an economic development drive that included the establishment of the world’s low-cost export base and its own domestically focused manufacturing industry (Young and Lan 1997). For 20 years, due largely to its vast, under-utilized rural labor supply that had languished for decades in remote rural communities, China was able to attract tens of millions (indeed hundreds of millions) of rural workers into low-cost manufacturing (Lu and Xia 2016). Throughout the 1980s and early 1990s, these labor-intensive workers, seen in China’s factories and on its construction sites, earned wages that were quite low when viewed in an international context (Yang et al. 2010). Because the rural economy employed large numbers of workers who had been engaged in low-productivity farming and sideline activities, China did not experience a significant rise in the labor-intensive wage rate during those first two decades (Lin et al. 1996). Indeed, in part based on this, China was able to launch its economy into one of the fastest periods of growth in history (Wei et al. 2017).

Although wages were still relatively low at the beginning of a new phase of development that emerged in the late 1990s and early 2000s, rising wages began to be a defining feature of China’s economy. Wages began to rise around the late 1990s (Yang et al. 2010), and, as the available supply of young rural workers who had not yet left the farm began to decline (de Brauw et al. 2002), wage growth gradually accelerated, beginning in the mid-2000s (Yang et al. 2010). Because the level at which these labor-intensive wages began in this period of increase was so low, at first, there was almost no noticeable change in the ways that firms organized themselves (Zhou and Tyers 2019). Gradually, however, firm managers and business owners began to look for ways to cut costs and improve profits (Hu and Kahn 1997). Indeed, in the early 2000s, growth continued in no small part due to labor-intensive technologies and business practices (Yueh 2015). Between 2003 and the early 2010s, China enjoyed what is now known to be its fastest period of growth. As employment opportunities were widely available, and wages trended upward, this growth period improved the welfare of labor-intensive workers throughout the country (Li et al. 2012a; Zhang 2018).

Due to the two distinct paths of employment and wage growth noted above, China’s growth pattern between 1980 and 2010 has been described as following the classical Lewis/Fei-Ranis model of economic development (henceforth, the *Lewis model*; Cai and Wang 2010). Using the lens of the Lewis model, we see that, in the early years as industrialization began, labor was pulled out of what was, at the time, an effectively unlimited pool of under-utilized workers in the rural population. In these early years, there was almost no effective increase in the labor-intensive wage rate. The shift from farming to off-farm employment with a stagnant wage rate lasted until the late 1990s, reflecting the first phase of the Lewis model (de Brauw et al. 2002). In the early 2000s, however, after large shares of the younger cohorts of both men and women already had moved into the off-farm sector, the unlimited supply of labor disappeared (Cai 2010; Knight et al. 2011). It was at that time that wages began increasing, gradually at first but then steadily through this period, following the path predicted by the second phase of the Lewis model (Cai and Wang 2010). These forces of supply and demand predicted by the Lewis model were also buffeted by policy change in the early 2000s, which contributed to the wage growth seen in China during that time (Liang et al. 2016; Liu et al. 2018; Lu and Xia 2016). As the Lewis model predicted, these two growth phases boosted China out of low-income status and facilitated its transition into a middle-income economy.

Although China did seem to follow the path predicted by the Lewis model over the first three decades of its era of reform, it appears that, in 2010, China reached another turning point in its economic development. As wages shifted ever higher during the 2010s, pressure began to build on some sets of firms, especially those that had been involved in low-wage/labor-intensive production activities, to make more fundamental adjustments to their business strategies. In some cases, wages became sufficiently high to motivate foreign firms that were producing for export to move their operations to other countries (Dawn.com 2019). A new trend of outward foreign direct investment by China’s firms also began to emerge in this same period (Mumtaz and Smith 2018). Such trends were, in part, responsible for the prominent rise of new electronic production bases in Vietnam (Martin 2018), the rapid expansion of textiles in Bangladesh (Emont 2019), and the relocation of many shoe manufacturers to Ethiopia, among other examples (Bain 2018). Cheng et al. (2019) also have documented the recent push of firms in China in the 2010s to automate to reduce the reliance on increasingly expensive workers and to avoid moving overseas.

This new phase of development that China has begun to enter, however, appears not to be unique to China. There is a subset of modern economies that, over the past several decades, have also experienced wage growth high enough to induce a fundamental shift in the nature of the technologies that firms use and the role of labor in the production process. The best known of the countries that most recently have moved successfully into this new phase are South Korea, Ireland, and Singapore (Li et al. 2017). In these countries, after wages were sufficiently high, firms began to globalize and automate to replace expensive labor, causing demand for labor-intensive labor inside firms to fall sharply. In the countries that have made this transition successfully, the workers who once worked on the factory floor or construction sites in labor-intensive jobs were able to transition into jobs with higher skill requirements (Rozelle and Hell 2020). The ability of the labor forces in these economies to make this transition depended on their human-capital levels, which were all relatively high at the time of this shift, allowing workers to make this transition (Khor et al. 2016). Importantly, after making these shifts, these economies’ growth continued, and wages continued to rise in most segments of the labor force. It is in this way that countries have broken through the high-wage/skill-intensive barrier, becoming nations that provide high and rising remuneration for a large share of their workforce.

Due to the composition of China’s labor force, the consequences of entering this third phase of employment and overall economic development in China are not clear. To the extent that its workers can adapt to these changes, globalization and rapid automation could help China to flourish. The level of education of the labor force in China, however, is systemically lower than that in South Korea, Ireland, and other successful middle-income “graduates” (Bai et al. 2019; Li et al. 2017). China’s workers, in general, have relatively weak skills, and their levels of education are, on average, low (Rozelle and Hell 2020). A growing service sector exists in China, but if the supply of labor-intensive workers who shift to the service sector outpaces demand for these services, fundamental questions about future wage trends arise.

The goal of our study is to examine the extent to which this third phase of economic development is impacting China’s economy. We seek to achieve our goal through addressing three specific objectives. First, we identify trends in employment data, generally and in terms of individual industries and sub-economies. Second, we examine wage data, also identifying trends at both the general and industry/sub-economy levels. Finally, drawing on the international literature on China’s recent economic development, we posit several explanations for the trends that we find. We include an examination of how wage polarization has played out in other economies—both middle income and high income—and the overall consequences for longer-term growth and social welfare in those economies.

The organization of our paper is as follows. In the next section, we describe the sources of the data that we examine. In the third and fourth sections, we identify employment and wage trends in that data. We then posit explanations for these trends in the fifth section. In the sixth section, we discuss wage polarization and potential consequences for China. In the seventh section, we conclude.

## Data

The data used in this paper are aggregated at the national level, and most are annual data published by the National Bureau of Statistics of China (NBSC). The one exception is data on migrant workers, which are drawn from the Annual Report of Monitoring Survey on National Rural–Urban Migrant Workers from 2010 to 2018 (NBSC 2010–2018).

Employment and wage data published by the NBSC are collected by several departments, each with distinct data collection approaches. One approach relies on the census of demographic changes, which directly surveys individuals about their employment information. Henceforth, we refer to this as *census data* or *census*-*based data*. The other data are a survey organized by China’s Bureau of Industry and Commerce that collects employee information via employers, henceforth, *employer survey data*. The census provides statistics on total employment, whereas the employer-based survey also provides information on employment and industry wage trends, including disaggregated data by sub-economy. This paper uses a combination of the data obtained by both of these NBSC-based data collection approaches.

In our analysis, we examine the data in several ways. When examining employment trends, we first look at total employment, which is measured by the census. We break down total employment into primary, secondary, and tertiary sectors, also using census data. When focusing on urban employment, we use data from both the census and the employer survey. When we break down urban employment and examine employment trends *by industry*, however, we use the employer survey data. In one part of the analysis, we focus our attention on several key industries: manufacturing, construction, labor-intensive services, and skill-intensive services. In another part, we break down urban employment into an alternative dimension: by employment in China’s formal and informal sub-economies. The data that allow us to examine China’s labor force by formal and informal sub-economies also are from the employer survey. All wage data, excluding that on rural–urban migrant workers, are taken from the employer survey. Rural–urban migrant worker employment data are taken exclusively from the Annual Report of Monitoring Survey on National Rural–Urban Migrant Workers.

## Employment Trends

### Overall Trends

Overall, total employment growth has been slow in recent years, with a few key exceptions. Table 1 shows that, in 2018, total employment (Column 1) slightly decreased for the first time since statistics were collected and reported (2004). Table 2 illustrates that growth of total employment (Column 1) was negative in 2018, although the growth rates of total employment have been very low (less than 1%) since the mid-2000s.

**Table 1:** Total employment trends in China, 2004–2017. *Sources* Total and urban employment data are from the National Bureau of Statistics of China (www.stats.gov.cn). Rural–urban migrant worker data are from NBSC’s Annual Report of Monitoring Survey on National Rural–Urban Migrant Workers (NBSC, 2010–2018)

Year	Total employment (census data)	Urban employment (census data)	Urban employment (employer survey)	Rural–urban migrant employment
2004	743	273	166	
2005	746	284	176	
2006	750	296	187	
2007	753	310	199	
2008	756	321	209	225
2009	758	333	224	230
2010	761	347	236	242
2011	764	359	266	253
2012	767	371	284	263
2013	770	382	325	269
2014	773	393	351	274
2015	775	404	370	277
2016	776	414	386	282
2017	776	425	403	287
2018	776	434		288

Employment in millions of individuals

**Table 2:** Total employment growth rates in China, 2004–2017. *Sources* Total and urban employment data are from the National Bureau of Statistics of China (www.stats.gov.cn). Rural–urban migrant worker data are from NBSC’s Annual Report of Monitoring Survey on National Rural–Urban Migrant Workers (NBSC, 2010–2018)

Year	Total employment (census data) (%)	Urban employment (census data) (%)	Urban employment (employer survey) (%)	Rural–urban migrant employment (%)
2004	0.72	4.05		
2005	0.52	4.02	6.18	
2006	0.44	4.37	5.90	
2007	0.46	4.47	6.61	
2008	0.32	3.72	5.07	
2009	0.35	3.80	6.86	1.93
2010	0.37	4.10	5.49	5.42
2011	0.41	3.54	12.56	4.36
2012	0.37	3.31	7.10	3.89
2013	0.36	3.07	14.27	2.41
2014	0.36	2.80	8.16	1.86
2015	0.26	2.80	5.40	1.28
2016	0.20	2.52	4.20	1.53
2017	0.05	2.50	4.46	1.71
2018	− 0.07	2.25		0.64

Growth rates are calculated by the authors

Although growth of total employment remained essentially stagnant, urban employment rose steadily (Table 1). According to the census data, urban employment expanded from fewer than 300 million workers in 2004 to more than 400 million workers in 2018. During this same period, the employer survey data show that although urban employment started at a level lower than was reported by the census in 2004, it also trended upward at a higher rate. These two trends also clearly demonstrate that, after 2012, the urban employment numbers reported by these two sets of data have begun to converge.

In terms of trends, Tables 1 and 2 show growth rate data that demonstrate that China’s urbanization is advancing. As seen in Table 2, the growth rate of urban employment continues to be higher than the growth rate of total employment, although urban employment growth rates also have begun to decline in recent years. Importantly, these trends hold for both the census-based and employer survey-based data, despite the fact that the points of decline begin at different points in time. The urban employment growth rate, as measured by the census, began to slow in 2010. This growth rate, as measured by the employer survey, although starting later, also shows that urban employment growth began to slow in 2013. Despite these declines in growth rates, by the late 2010s, the growth rates of urban employment were still significantly higher than those of total employment (which even became negative in 2018).

### Employment Trends by Sectors and Industries

Although overall employment reached a peak and urban employment growth rates have begun to gradually slow down (in both data sets), when looking at individual sector and industry data, we see distinctly different growth trajectories. As Table 3 shows, in 1999, the primary sector (which comprises mainly individuals who are engaged in agricultural work) accounted for around half of all employment, followed by the tertiary and secondary sectors. Since 2002, however, employment of the primary sector has fallen rapidly. In 2011, primary sector employment was lower than that of the tertiary sector, and, in 2014, it was surpassed by the secondary sector. Since then, the employment share of the primary sector has continued to decline.

**Table 3:** Total employment trends in China by Sector, 1999–2017. *Source* National Bureau of Statistics of China (www.stats.gov.cn)

Year	Employment	Employment in primary sector	Employment in secondary sector	Employment in tertiary sector
1999	714	358	164	192
2000	721	360	162	198
2001	728	364	162	202
2002	733	366	157	210
2003	737	362	159	216
2004	743	348	167	227
2005	746	334	178	234
2006	750	319	189	241
2007	753	307	202	244
2008	756	299	206	251
2009	758	289	211	259
2010	761	279	218	263
2011	764	266	225	273
2012	767	258	232	277
2013	770	242	232	296
2014	773	228	231	314
2015	775	219	227	328
2016	776	215	224	338
2017	776	209	218	349

Employment calculated based on census data

Employment in millions of individuals

In contrast to the primary sector, employment in the secondary sector (which includes both manufacturing and construction) was mainly rising until more recently (Table 3). From the early 2000s through the early 2010s, employment in the secondary sector experienced steady growth, reaching a peak of 232 million in 2012. After 2012, however, this trend reversed, and employment in the sector began to fall, continuing to do so even today. In 2017, only 218 million workers were working in the secondary sector.

The tertiary sector, in contrast, has been growing steadily over the entire survey period (1999–2017) and has accelerated in recent years (Table 3). Before 2011, although employing a modestly higher number of workers than did the secondary sector, tertiary sector employment (including labor-intensive services as well as more high-skilled employment in certain sub-industries, such as high technology, banking, and education) was still below the primary sector. After 2011, however, employment in the tertiary sector surpassed that of the primary sector, becoming the sector in China’s economy with the highest level of employment. With the increase in employment of the tertiary sector and the simultaneous decrease in that of the primary and secondary sectors, the gap in the number of workers employed between the three sectors has become increasingly wide.

Trends in urban employment are similarly varied, largely following those of total employment. According to the employer survey, urban employment includes both major secondary (including manufacturing and construction) and tertiary industrial sectors (including labor-intensive and skill-intensive service sectors). As seen in Table 4, overall urban employment in the manufacturing and construction industries began to decline after 2014, in keeping with the overall trend of the secondary sector (Table 3). In contrast, the data illustrate that urban employment in the service industry has been growing. According to the employer survey data, which allows a division of the employment data for overall services in the urban sector of the economy into employment in labor-intensive and skill-intensive services, employment in both industries was rising between 2004 and 2017. Importantly, throughout this period, and especially after 2010, the increase in the number of workers in the labor-intensive service sector has been accelerating.

**Table 4:** Urban employment in China by Industry, 2004–2017. *Source* National Bureau of Statistics of China (www.stats.gov.cn)

Year	Manufacturing	Construction	Labor-intensive services	Skill-intensive services
2004	42	11	52	62
2005	45	12	56	64
2006	49	13	60	65
2007	52	14	66	68
2008	53	15	71	70
2009	55	16	80	73
2010	58	18	84	76
2011	64	23	98	81
2012	66	26	105	87
2013	77	36	119	93
2014	80	37	137	98
2015	78	37	153	102
2016	77	37	165	106
2017	76	37	180	111

Employment calculated based on employer surveys. Labor-intensive services include transportation and storage; wholesale and retail trades; hotel and catering services; leasing and business services; and services to households and other services. Skill-intensive services include information; transmission, computer services, and software; financial intermediation; real estate; scientific research; technical services and geologic prospecting; education; health care and social welfare; and culture, sports, and entertainment

Employment in millions of individuals

### Employment Trends in the Formal and Informal Economies

By breaking down employment by sub-economy (that is, those employed in the formal and informal sub-economies), the data reveal another set of trends. As Table 5 illustrates, employment in the formal and informal sub-economies has largely traded places. In the early 2000s, formal employment accounted for more than 65% of total employment (Row 1, Column 3). Since then, employment in the informal sector has risen, and, in 2017, it accounted for just under 60% of all employment (Row 14, Column 3). The steepest rise in informal employment began in 2013 (Row 10, Column 3), as did the steepest fall in formal employment.

**Table 5:** Urban formal and informal sub-economy employment in China, 2004–2017. *Source* National Bureau of Statistics of China (www.stats.gov.cn)

Year	Formal employment	Informal employment	Formal employment share of total employment (%)
2004	111	55	66.80
2005	114	62	64.65
2006	117	70	62.70
2007	120	79	60.38
2008	122	87	58.27
2009	126	98	56.23
2010	131	105	55.33
2011	144	121	54.28
2012	152	132	53.58
2013	181	144	55.73
2014	183	169	52.01
2015	181	190	48.76
2016	179	207	46.34
2017	176	227	43.76

Employment calculated based on employer surveys. Employment in millions of individuals

With a focus on industries within sub-economies, Panels A and B of Table 6 reveal the main forces behind the trends in China’s overall employment shifts. In terms of the formal sub-economy, the fall in the share of formal employment (Table 5) is driven by falling employment in the manufacturing and construction industries (Panel A). Skill-intensive services in the formal sub-economy, however, continue to grow steadily. In the informal sub-economy, the main driver of employment is the labor-intensive services sector (Panel B). The shares of employment in the other informal industries (manufacturing, construction, and high-skilled services), even when added together, account for only 28% of informal employment. Hence, when discussing informal employment in China, one is actually talking about labor-intensive services.

**Table 6:** Industrial Structures of Employment in the Formal and Informal Sub-economies in China, 2004–2017. *Source* National Bureau of Statistics of China (www.stats.gov.cn)

Year	Panel A (formal employment)				Panel B (informal employment)			
	Manufacturing	Construction	Labor-intensive services	Skill-intensive services	Manufacturing	Construction	Labor-intensive services	Skill-intensive services
2004	31	8	16	56	12	2	35	6
2005	32	9	16	57	13	2	40	7
2006	34	10	16	58	16	3	44	7
2007	35	11	16	59	18	3	49	9
2008	34	11	17	61	19	4	55	10
2009	35	12	17	63	20	4	63	11
2010	36	13	17	65	22	5	66	12
2011	41	17	19	68	23	6	79	14
2012	43	20	20	70	24	6	85	17
2013	53	29	25	75	25	7	94	19
2014	52	29	26	76	27	8	111	23
2015	51	28	26	77	28	9	127	26
2016	49	27	26	78	28	10	140	29
2017	46	26	26	79	29	10	154	33

Employment calculated based on employer surveys. Labor-intensive services include transportation and storage; wholesale and retail trades; hotel and catering services; leasing and business services; and services to households and other services. Skill-intensive services include information; transmission, computer services, and software; financial intermediation; real estate; scientific research; technical services and geologic prospecting; education; health care and social welfare; and culture, sports, and entertainment

Employment in millions of individuals

### Summary of the Characteristics of Employment Trends

Since the early 2000s, although total employment has remained fairly steady, trends in employment by economy, sector, and industry have fluctuated, and notable trends have emerged. There has been a sharp rise in employment in the informal sub-economy, while employment in the formal sub-economy has stagnated (and, in fact, fallen from 181 million workers in 2013 to 176 million in 2017). In contrast, during the same time period (2013–2017), employment in the informal sub-economy rose from 144 million workers to 227 million.

These trends in the formal and informal sub-economies also have industry-specific drivers. In the formal sub-economy, there has been a fall in the absolute number of workers employed in the manufacturing and construction industries (starting in 2013), while employment in the skill-intensive service industry has continued to rise. In the informal sub-economy, employment in the labor-intensive service industry rose from 2004 to 2017, the entire period covered by our data, and accelerated between 2013 and 2017. These trends have been occurring over the last decade, and the most prominent employment trend shifts occurred in 2012 and 2013 in both the formal and informal sub-economies, which may suggest that China is beginning to move into a new phase of growth. To further explore this possible transition, we examine trends in wage growth throughout the Chinese economy.

## Wage Trends

Although wages across industries have continued to rise since 2000, the trends of these wages across different types of employment have begun to diverge. Table 7 illustrates the divergence of wages of individuals in the formal and informal sub-economies through differences in the rates of wage growth in the two sub-economies. According to data from the employer survey, the growth rates of wages in both sub-economies have fallen between 2010 and 2017. The table shows that the rate of decline of the growth rate of informal sub-economy wages is greater than that of the formal sub-economy. In fact, whereas before 2014, the growth of wages in the informal sub-economy was higher than those in the formal sub-economy, after 2015, the growth of informal sub-economy wages fell below that of the formal sub-economy. Looking at this another way, by 2016, the growth rate of wages in the informal sub-economy was slower than China’s overall GDP, while the growth rate in the formal economy was growing faster than the GDP.

**Table 7:** Real growth rates of the average annual wage and GDP in China, 2010–2017. *Source* National Bureau of Statistics of China (www.stats.gov.cn)

Year	Formal wage growth (%)	Informal wage growth (%)	GDP growth (%)
2010	9.70	10.42	10.60
2011	8.53	12.23	9.60
2012	9.05	14.12	7.90
2013	7.29	10.87	7.80
2014	7.33	9.08	7.30
2015	8.54	7.29	6.90
2016	6.80	6.07	6.70
2017	8.26	5.15	6.80

The real growth rates of wages are calculated using the same data as those of Table 6 and are reduced by CPI

To illustrate the composition of the informal sub-economy, Table 9 in the Appendix provides a comparison of the annual wages of the informal sub-economy with the annual wages of rural–urban migrant workers. We also replicate Table 7 in the Appendix, but, instead of using formal sub-economy wages, we substitute the growth rate of annual wages (in Appendix Table 10) of those in the formal economy with the growth rate of annual wages of rural–urban migrant workers. Overall, the wage and wage-growth trends seen in the tables (Table 7/Appendix Tables 9 and 10) are nearly identical. Thus, when one is talking about individuals in the informal sub-economy, one could easily substitute the term *rural*–*urban migrants*.

This divergence between wages in the formal and informal sub-economies is seen in Table 8, which provides wage trends by skill type. The trends among labor-intensive workers and skill-intensive workers mirror those seen in Table 7 (when we compare wage trends in formal and informal sub-economies). Between 2010 and 2014, wage growth in informal labor-intensive industries (10.68%; Row 1, Column 2) was higher than that of formal skill-intensive industries (7.75%; Row 1, Column 1). Between 2015 and 2017, however, the wage-growth rate of the labor-intensive industries (6.02%; Row 2, Column 2) was lower than that of the skill-intensive industries (9.07%; Row 2, Column 1). Overall, during this period of data availability between 2010 and 2017, while the growth rate of wages in the skill-intensive industries was rising, the growth rate of wages in the labor-intensive industries was falling.

**Table 8:** Average real growth rates of wages in China by Skill type. *Source* National Bureau of Statistics of China (www.stats.gov.cn)

Years	Average annual growth rate	
	Formal skill-intensive wages	Informal labor-intensive wages
2010–2014	7.75%	10.68%
2015–2017	9.07%	6.02%
Change (percentage points)	1.32	− 4.66

Employment calculated based on employer surveys. Labor-intensive services include transportation and storage; wholesale and retail trades; hotel and catering services; leasing and business services; and services to households and other services. Skill-intensive services include information; transmission, computer services, and software; financial intermediation; real estate; scientific research; technical services and geologic prospecting; education; health care and social welfare; and culture, sports, and entertainment

### Summary of the Characteristics of Wage Trends

Although wages across all sectors, industries, and sub-economies have a variety of trends, when we consider some of the largest and highest-profile industries in recent years, we can see two major sets of trends. First, the growth rate of wages in the formal sub-economy (which, post-2014, is also higher than the growth of GDP) has become higher than that in the informal sub-economy (which is now lower than the growth of GDP). Second, in terms of wage performance over the past decade in terms of skill differences, while the rate of wage growth has been slowing for those workers in low-skilled industries, wage growth has been increasing for high-skilled workers. Although we do not have the raw data to test this hypothesis formally, the trends indicate that the divergence of both wage levels and the rates of wage growth between the formal and informal economies are being driven, in no small part, by the emergence of the polarization of wages between those in the low-skilled and high-skilled sectors.

Moreover, taking employment and wage changes together, we can see that employment and wage trends in China have involved two divergent sub-economies in recent years. We see that, for skill-intensive industries in the formal sub-economy, there were modest increases in employment but relatively strong wage-growth rates (especially for a country in which the overall growth rates are falling). In contrast, for labor-intensive industries in the informal sub-economy, there has been a sharp increase in employment (much more rapid than the increases in skill-intensive industries in the formal sub-economy). Unlike skill-intensive workers in the formal sub-economy, labor-intensive workers in the informal sub-economy have suffered a decline in wage growth. That these transitions began to occur almost simultaneously around 2013 may indicate that China’s economy has entered a new phase of economic development. We still need to understand, however, why China’s industries have exhibited divergent growth in this new phase, which we address in the section below.

## Why Employment and Wages Exhibit Divergent Trends

As shown in the previous section, the polarization of wages amid rising employment has been observed in China’s formal and informal sub-economies. The acceleration of the divergence in wages in the presence of growth in employment in both sub-economies appears to be driven by the employment decisions of individuals who are employed (or are destined to be employed) in China’s labor-intensive and skill-intensive industries. In this section, we identify and discuss some of the major determinants of the demand for and supply of labor in each of these sub-economies to better understand the asymmetric trends between the wages of skill-intensive and labor-intensive workers. Here, we rely exclusively on reviews of the literature and do not have primary analyses that use data-based empirical evidence due to an absence of appropriate data.

### Skill-Intensive, High-Wage Service Industry^1^

In this subsection, we examine the rise of employment and wages in China’s formal sub-economy’s skill-intensive service industry. To better explain how employment and wages can both be rising, we break down, below, these trends into two categories: the demand for and supply of skill-intensive workers. Our objective is to show that the relative rates of the expansion of the demand for skill-intensive labor (relatively higher) and the supply of skill-intensive labor (relatively lower), as explained through the review of the literature, lead to a set of trends in the skill-intensive industry of the economy: rising employment and rising wage (levels and growth) in the skill-intensive service industry.

#### Skill-Intensive Service Demand

We begin by examining the growth of demand for skill-intensive workers. As China’s GDP has grown and its economy has become richer, demand for employment in skill-intensive sub-industries, such as high technology, finance, education, and health care, has increased. Particularly representative of this growth is the high-technology industry, which, in the early 2010s (between 2011 and 2017), saw tremendous growth in the number of firms, adding nearly 8% annually to the ranks of domestic companies (Wong 2020). Even more evident has been the rise of large and high-profile, high-technology firms, with Alibaba and Tencent in the top 50 of the world’s largest companies (Murphy et al. 2020). With the growth of small firms (in numbers) and large firms (to very large sizes), China is now home to one of the most vibrant high-technology industries in the world (Charlton 2019).

Other high-skilled industries have had similar growth paths. China’s banking system has more than doubled in size between 2010 and 2017 (Spross 2018). China’s stock market, worth under 10% of GDP in 1997, has grown to over 60% of GDP in 2016 (Naughton 2018). Public expenditure on education, especially higher education, also has more than doubled from 2011 to 2019 (Textor 2020a). Likewise, both the health industry and insurance sub-industry have seen compounded double-digit growth between 2015 and 2020 (Zeng et al. 2020). The expansion of these sub-industries (among others) is an important facet of the growth in demand for skill-intensive workers.

We also examine what is behind the rapid growth of these skill-intensive jobs. In one sense, China is simply following the path of other middle-income nations that have seen demand for employment in skill-intensive industries rise. The history of economic structures in other middle-income countries includes sharp rises in similar sub-industries during periods of economic expansion. For instance, the Argentinian technology sector saw exceptional growth during the economic boom between 2003 and 2013, with government spending on R&D that nearly doubled between 2008 and 2013 (UNESCO 2015). The South African experience is similar in the financial sector, as the total assets held by financial institutions nearly quintupled between 2002 and 2018 (Rudden 2020). China’s experience today mirrors these trends.

In addition to following the income-led rises in the demand for the goods and services in China’s high-skilled industries, a set of government programs have pushed growth of high-skilled industries in China’s economy. The growth in the high-technology sector, for example, although to some extent tied to consumer demand, also was facilitated by government investment in R&D. The percentage of GDP that China’s government has dedicated to R&D climbed from 1.7% in 2010 to 2.2% in 2017, which is almost as high as that of the USA (2.8% in 2017) and far surpasses that of other middle-income countries, such as Brazil (1.3% in 2017), South Africa (0.8% in 2017), and Mexico (0.3% in 2017) (“Science & Technology” 2020). R&D expenditures almost certainly had impacts on the rise of many of the high-skilled industrial sectors, and funding specifically for the high-technology sub-industry has been prioritized by China’s government (Charlton 2019; “Made in China 2025’ plan issued” 2025).

Other government-led efforts are targeted at supporting other sectors that demand large numbers of high-skilled workers. The 13th Five-Year-Plan, China’s primary planning policy instrument unveiled in 2016, demonstrates the government’s objective of investment into technology and other domestic industries, such as finance, education, and health care. In high technology, the plan outlines the government’s objective of making “breakthroughs in the industrial application of key technologies such as next generation photovoltaics, high-efficiency, high-wattage wind power generation,” among other high-technology applications. In finance, the plan allocates hundreds of billions of RMB from China’s banks to be opened as credit lines for firms to pursue modernization and development plans (Central Committee of the Communist Party of China 2016). Other policy initiatives have encouraged foreign firms to invest in certain sub-industries, many of them with high-skilled intensive worker demand, and this has brought into China increasing FDI inflows (“Foreign Investment in China” 2020; “Foreign Direct Investment, Net Inflows” 2020). Government initiatives also have played key roles in the recent growth and continued expansion of the high-skilled sectors of China’s economy.

The above factors have contributed to the rise of skill-intensive worker demand, and of special note is the pursuit of automation by China’s government, which could have a great effect on increasing the demand for high-skilled workers as well as dampening the demand for low-skilled manufacturing workers. In fact, there is no country that has seen the pace of automation rise faster than has China (Cheng et al. 2019). According to Zhou and Tyers (2019) and the Development Research Center of the State Council and the World Bank Group (2020), automation sharply increases the human capital required for manufacturing jobs, which, in turn, increases employment for skill-intensive workers. For instance, China’s growth trend and projected adoption of over one million industrial robots in 2020 will require over 200,000 skill-intensive practitioners to maintain their successful operation (Cheng et al. 2019; Xiao 2020). These trends are boosted directly by the Made in China 2025 plan, which calls on firms in China to use “automated processes” and “intelligent manufacturing”—robots—in their development plans (Bateman 2018).

China’s progress as a middle-income country has triggered surging demand for skill-intensive sub-industries. Indeed, high growth in the past decade in the sub-industries of high technology, finance, education, and health care are indicative of the overall trend in China toward skill-intensive worker demand as incomes rise. Although, to a certain extent, this growth mirrors the international precedent of middle-income growth, unique programs and incentives, such as the Made in China 2025 plan, have boosted the growth in the demand for skill-intensive workers in China. In summary, then, the combination of rising incomes (and rising demand for products in skill-intensive sub-industries) and government incentives has led China’s demand for skill-intensive workers to rise in recent years.

#### Skill-Intensive Service Supply

Now that we have established that there has been a steady rise in demand for skill-intensive workers, we focus on issues that may be affecting the supply of skill-intensive workers to illustrate the relationship between supply and demand in the current trend of rising wages. We first examine the nature of the main channels of the supply of labor to the high-skilled sectors of China’s economy. To do so, we focus on the higher education system and its recent record in producing graduates. The initial findings are that China is producing a substantial number of college graduates, which, prima facie, could mean that the rise in the supply of high-skilled labor is exceeding the rise in demand, which, as noted above, is still rising steadily. Then, we discuss the findings in the literature that show why the rise in the supply of high-skilled workers (who are able to be effectively employed in the skill-intensive sector) may be lower than presumed, at least in recent years.

As noted, the number of college-educated workers in China has grown dramatically in the past several decades. In 1999 alone, the Chinese government increased the admissions quota by 43%. Between 1999 and 2005, China’s college admissions quadrupled, and between 1999 and 2009, the average annual growth rate of college admissions was 18%. By 2015, 13% of China’s urban labor force had a college degree (Li et al. 2017), and, in 2010, more than 60% of high school students attended a university upon graduating (Levin 2010).

Due in part to this expansion of higher education, China has become one of the world’s leading “producers” of students who earn science, technology, engineering, and math (STEM) degrees (Stapleton 2017). In fact, in recent years, 40% of China’s graduates in its ever-growing high education system are STEM majors, which is twice the percentage found in US schools (Schleicher 2016). In addition, many of China’s students are obtaining international degrees at US universities, with the number of students from China who hold US degrees at an all-time high. In 2018–2019, there were approximately 370,000 students from China who were studying in US universities, up from around 98,000 in 2008–2009 (Textor 2020b). It is evident that China is producing a much larger number of college graduates than it had previously, and it will be from this pool that the supply of workers for skill-intensive sub-industries will come.

That China is producing more college graduates in recent decades has led many to believe that China is over-producing them (Jacobs 2010), boosting the supply of skill-intensive workers to a level that might be expected to put downward pressure on skill-intensive wages. When more closely examining this issue, we can see that this is not necessarily the case. In fact, the literature suggests that the supply of skill-intensive workers in China may be overestimated, at least for the coming years. The size and quality of this new supply of college graduates who are employable in the skill-intensive sector is frequently misunderstood because starting wages for college graduates are often the same as (or even lower than) those of labor-intensive workers (Li et al. 2012a, b). This perception of there being too many graduates who are ready to enter the skill-intensive sector is further called into question by studies that show that, in the months that follow graduation, college graduates in China often find themselves unemployed (Gu 2013; Li et al. 2017). Low starting salaries and lag times between graduation and employment, however, cannot be used summarily as a metric to lead to the conclusion that there is an oversupply of college graduates who are ready to enter skill-intensive employment (Jacobs 2010). In fact, the literature indicates that once college graduates have begun to acquire experience, the return to college education begins to appear (Li et al. 2012b). These trends also could be reinforced by the positive externalities that are created by rising human capital (Glaeser and Lu 2018; Liang and Lu 2019).

Moreover, the data indicate numerically that the college graduate supply is not oversaturated. In fact, despite the advances that China has made in enrolling students into tertiary education, China’s census shows that, in 2010, only 12.5% of the overall labor force was college educated, lower than that of most other middle-income countries (Li et al. 2017; NBSC 2010). In other words, there is a large and growing supply of educated workers who have the potential to enter China’s skill-intensive sub-industries; this, however, is limited to graduates with sufficient experience. One interpretation of skill-intensive employment/wage trends is that the supply of educated and experienced skill-intensive workers has been growing but that the supply has not kept pace with demand, leading to rising employment and rising wages in this sub-industry.

### Labor-Intensive Service Industry^2^

We now examine the opposing set of trends (of rising employment and falling wages) in the informal labor-intensive service sub-industry. First, we examine some of the forces that are creating demand in the service industry for low-wage, labor-intensive jobs. Then, we examine the factors that influence supply. Given the observed trends that employment is rising and wages are falling, we pay attention to issues that appear to be restricting demand for service-industry labor and accelerating supply of that labor.

#### Labor-Intensive Service Demand

Here, we first look at the rise in the demand for labor in the low-wage, labor-intensive service industry. To do so, we present the international precedent for the demand for labor-intensive services in developing countries, in which we witness rising GDP as leading to a growing demand for services through increased income available for consumption. We then explore investment and demand for government services that we believe reduce the share of GDP available for consumption. We also examine how high savings by consumers, which are associated with high levels of risk in the economy, further reduce the income available for consumption. Of course, further shrinking of GDP available for consumption would naturally curtail demand for informal services by Chinese consumers, and we explore the notion that if large shares of GDP are devoted to areas other than consumption, service demand may not be able to expand quickly enough to absorb all of the incoming labor supply, which would result in our observations of falling labor-intensive service-industry wages amid rising employment.

In most economies in the world, as GDP grows, the demand for services rises (Gustafsson et al. 2020). The international precedent in nations that are in the process of growing from middle-income toward high-income status is that this period is typically one of robust growth of a service industry (Kharas and Kohli 2011). Indeed, a study by Diacon and Maha (2015) illustrates how, as GDP rises in most countries that have reached upper-middle-income status, there is a strong correlation with these increases in income, which enhances the buying power of the population and creates a growing demand for services. Hence, given China’s high rates of GDP growth over the past decade, one would expect that there would be a very large expansion in the demand for services in the economy.

Although there has been a significant increase in the demand for services in China during recent years, there are two related factors that may be cutting into the GDP available for consumption and, by extension, services in China. The first limit on the share of GDP that may be driving the demand for services is the government’s focus (from the earliest days of the People’s Republic) on a growth strategy based on high levels of investment (Naughton 2018). Indeed, to the detriment of other areas of the economy, China’s government has devoted large portions of GDP toward physical capital and other forms of investment (Heckman 2005). Investment accounted for 43% of China’s GDP in 2019 compared to 20% of GDP for the USA, 21% for Mexico, and 15% for South Africa at the end of the same year (“China Investment:  % of GDP [1952–2020]” 2020). Over the past decades (including in the 2010s), the allocation of these funds has been focused on pursuing a capital-intensive, investment-based growth strategy in China (“China Capital Investment, Percent of GDP” 2020). As a consequence of this investment-first strategy, there has been a lower share of GDP available for overall consumption and, by extension, less demand for services.

Further shrinking the share of GDP that is available for consumption is the large share of funds used to support government services, including to support salaries and other expenses associated with China’s large state bureaucracy. In recent years, expenditures by China’s government accounted for more than one-third (i.e., 34% in 2019) of GDP (“Fiscal Monitor (April 2020)—Expenditure” 2020). These numbers are much higher than those for South Africa, another BRICS (emerging national economy) country, which spent just over 20% of GDP in 2018 (“South African Government Spending, Percent of GDP” 2020). In part, a large share of China’s GDP is used to support the largest bureaucracy in the world. Over the past decade, China’s public employment was reported to be 77.6 million people, constituting 10.2% of China’s total labor force (Rutkowski 2013). The operational expenses of this government, including the programs run by and payrolls for the sizable public service population, absorb a large portion of GDP redirected from uses that could otherwise boost consumer spending on services.

In addition to the sizable portion of GDP spent on China’s formal government bureaucracy, a large share of GDP is also dominated by China’s State Owned Enterprises (henceforth, SOEs), which, given the focus of the business activities of China’s SOEs, means that there is a capital-intensive/investment-oriented bias. This important role of SOEs is seen in a number of areas. In the late 2010s, the output of SOEs accounted for roughly 30% of GDP (Zhang 2019), and around 30% of industrial assets are also owned by SOEs. Finally, national, regional, and local SOEs account for approximately 40% of the total enterprises in China. Moreover, most of this investment for the past several decades has been targeted toward capital-intensive (not labor-intensive) industries. Putterman and Dong (2000) and Zhang (2019) show that the composition of SOE investments is inordinately focused on electricity, oil and gas, steel and other metals, and transportation-related industries, among other investment-intensive industries. Finally, in addition to being a source of capital-heavy investment, SOEs are institutional tools for the central government’s development plan (Chen and Chen 2019; Jones and Zou 2017). Hence, the focus of government officials on the promotion of capital-intensive SOEs is part of a series of economic forces that combine to reduce the share of GDP that is in the hands of consumers.

Although the economy’s focus is on investment and government expenditures, in the past decade, there continued to be a growing middle class in China, and, as found in many other nations of the world, when the middle class is growing, often, there also is a robust rise in the demand for services. The expansion of China’s middle class, with increasingly higher levels of disposable income, has been one of the foundations of China’s economic push for the expansion of its service industry (Liao 2020). Notably, the rise in the middle class has been tremendous, from nearly zero in the 1990s to more than 400 million people in China today who can be said to be living at middle-income levels (Gustafsson et al. 2020).

To cater to the needs of this growing share of the market, service-industry firms in China, such as the ridesharing company Didi, have emerged. Due to the rapid growth of the demand for such services, since 2012, Didi has raised US $21 billion in 18 rounds of funding as of March 2020 (Ciaccia 2020). Another example of the rise of the service industry is the growth of another domestic firm, an in-home food delivery service known as Meituan Dianping. Even though it has many competitors, the rise in the demand for Meituan’s services since the early 2010s has resulted in the creation of nearly 20 million job opportunities (Lee 2019). Despite the fact that a large share of GDP is going toward investment and government services, China’s growth has been high enough to spur a sharp rise in the demand for labor in the service industry.

Nevertheless, there are other factors in China’s economy that are dampening this emerging demand for services. One such factor is the high rate of savings that characterizes China’s economy. Savings rates are high in economies when there is a feeling among consumers that they face relatively high levels of risk with little institutional insulation from this risk (Chamon and Prasad 2010). Among middle-income households, the households that should be driving the increase in the demand for services, the rate of savings is around 34% (Gustafsson et al. 2020; Zhang et al. 2011). In contrast, the USA has an average savings rate of 19%, and the rate of similarly middle-income Mexico is only 23% (“gross savings (% of GDP)” 2020). These high savings rates are diverting disposable income from raising demand for services.

The literature has identified several important factors that appear to motivate households to put aside sizable shares of their income into savings. First, the lack of a social safety net forces Chinese consumers to spend out of pocket to insulate themselves from institutional risks embedded in an economy with patchwork social services (“Socialism’s Precariate—A slump exposes holes in China’s welfare state” 2020; “China’s social security system” 2019; Dong and Cui 2010). Second, prohibitively high housing costs may be further pulling potential consumption income into savings, with some areas in China that have experienced more than a 300% increase in housing prices since the early 2000s (Feng and Wu 2015). High housing prices are made worse by the fact that there is considerable difficulty in gaining access to mortgage loans for many prospective home buyers. In fact, in addition to housing, there are high barriers to entry for acquiring any type of loan from the financial system, such that consumers often need to first save and then spend (Liu 2020). Thus, the high savings rates are the lack of a comprehensive social safety net, an adaptation to high housing prices, and poor access to financial systems (Chamon and Prasad 2010). These high savings rates are prominent throughout the economy, as the consumer constraints created by the Hukou system necessitate high savings rates and reduced consumption for the two- to three-hundred million migrant workers in China (Chen et al. 2015). These factors also mean that consumers necessarily will have smaller portions of their disposable income available for services.

#### Labor-Intensive Service Supply

We now focus on the factors and industries that appear to be contributing to the high levels of labor supply in the labor-intensive service industry. As noted in the previous section, falling wages are a symptom of a situation in which the supply of labor to the service industry is rising faster than is the demand for labor. Here, then, we examine this supply surge, identifying the sources of excess labor that spills into the service industry rather than flowing into other market industries. We focus on three potential sources of labor that may be moving into the service industry: agriculture, manufacturing, and construction. In each of these industries, we examine forces that may be stopping the flow of labor into other industries and/or releasing the flow of labor out of each of the alternative industries and into the service industry.

We begin with agriculture, which, in the 1980s and early 1990s, was a major outflow source of labor-intensive employment into the manufacturing and construction industries but has since tapered off. In the late 1990s and early 2000s, labor-intensive wages began to rise as China passed the Lewisian turning point, meaning that sources of excess labor from the agriculture industry had been largely depleted. In fact, as pressures in the labor-intensive workforce rose through the 2000s, these forces led to rising wages of labor-intensive workers in the manufacturing and construction industries, as the high wage rates were not able to draw significantly large numbers of additional workers out of agriculture (Cai and Du 2011; Wang 2010; Zhang et al. 2011). According to de Brauw et al. (2002), national samples show that, even by the mid-2000s, nearly all young rural individuals were working off the farm. Those working-age individuals who remained in rural areas were often middle-aged women whose tasks, in addition to farm work, included taking care of children and the elderly (Zhong and Xiang 2012).

In addition to the falling flow of labor from agriculture, there are a variety of reasons that agriculture does not attract labor from other industries. First, although nearly every rural family in China has access to farmland, average plot sizes in 2000 were extremely small, at around 0.5 hectare/capita (Ji et al. 2016). Due to these small sizes of farms, a person who works off the farm at current wage rates for one month can make more than what the average farmer makes on his or her family’s land in one year (Cheng et al. 2020). Moreover, even if that worker wanted to enter agriculture, the labor-force experience over the past 30 years indicates that the vast majority of rural workers lack the requisite farming skills (Chan 2010). Finally, there are few incentives to return from urban centers to agricultural rural areas, as job markets in these areas are limited (Gao et al. 2015). Looking forward, the agriculture industry, lacking the ability to attract new labor, does not appear to be a viable destination for workers.

Given that the pool of surplus labor from agriculture was depleted many years ago, the large flow of labor that we see entering the service industry needs to come from other sources, namely manufacturing. Although manufacturing in past decades was responsible for employing hundreds of millions of labor-intensive workers, two institutional shifts are fundamentally changing the direction of the flow of employment. First are the challenges faced by manufacturing due to globalization and international competition (Wolcott 2018). As labor-intensive wages rose after 2000, the labor costs faced by labor-intensive manufacturing firms in China led many companies to look for opportunities for manufacturing abroad (Tate et al. 2014). For example, in 2013, Samsung started planning a partial move of its factories out of China, with the hope of nearly completely relocating their manufacturing base (“Samsung Moves Factories from China to Vietnam” 2013). In 2015, Samsung had moved 40% of its manufacturing to Vietnam (Maierbrugger 2013), and, by 2018, only 20–30% of its manufacturing remained in China (Martin 2018).

Similar moves have occurred in the shoe industry. In 2012, over one-third of Nike’s shoes were produced in China, whereas, in 2017, that portion was reduced to less than 20% (Wolf 2018). Adidas, which, in 2007, produced more than 50% of their shoes in China, now produces less than 20% in China (Bain 2018). Many textile manufacturers have moved their operations to Bangladesh and other comparatively low-wage nations (“Chinese Textile Companies Shifting Production to Bangladesh” 2015; Emont 2019). In recent years, China has accounted for 11–30% of the production of many international textile firms, whereas, in the past, many firms were producing 30–50% of their textile manufacturing in China (Lu 2018). The rapidly changing environment of labor-intensive manufacturing, in particular, is likely a primary driver for the labor flow expansion to the service industry.

Recent international events and political forces suggest that globalization and demand fall for labor-intensive workers may be expected to continue or even accelerate. Starting in 2019, the US-China trade war incentivized manufacturing companies to begin reshoring, as supply-chain uncertainty and political pressure from US politicians made firms consider moving their operations outside of China (Emont 2019; Li 2019; Rapoza 2020; Van den Bossche et al. 2020). In the first half of 2019, China’s exports dropped by 25 billion dollars, which is said to have benefited mainly less-expensive suppliers in Asia (Colback 2020). The trade war also has been accelerating the shift of many apparel factories out of China. For example, Uniqlo, Levi’s, Crocs, Tommy Hilfiger, and Calvin Klein have all significantly decreased the amount of manufacturing in China after sanctions were imposed by the Trump administration (Twigg 2019). More recently, the political fallout from the COVID-19 crisis is expected to make reshoring out of China a far more immediate concern (Van den Bossche et al. 2020). Thus, even if the shift of factories out of China begins to have moderating effects on the level of wages of workers, for labor-intensive workers, the seeming instability of manufacturing in China will work to further reduce domestic Chinese demand for labor-intensive manufacturing workers.

A second fundamental reason for the outflow of labor-intensive workers from China’s manufacturing industry is automation, which, like globalization, has been shown to have displaced many workers in China’s economy. Automation of many industries in China is associated with concerns over rising wages (Cheng et al. 2019). When firms commit to large-scale automation programs, there are ripples throughout the manufacturing industry, as large numbers of labor-intensive workers within the industry are left without jobs (Paus 2018; Wolcott 2018). Government initiatives offer further incentives for companies to automate, as in the Made in China 2025 strategic plan, an initiative modeled after Germany’s Industry 4.0 manufacturing transformation (Paus 2018). This program pushes firms to automate and embrace high-technology avenues of production, as the Chinese government’s dedication to science, technology, and research in the area of technology-based industrialization has been supported by significant investments and low-interest loans (“Is China Ready for Intelligent Automation” 2020). Paradoxically, although increases in automation correlate with falling demand for labor-intensive workers and subsequent wage-growth reductions (Zhou and Tyers 2019; Development Research Center of the State Council and World Bank Group 2020), there appears to be no reduction in the government commitment to support large-scale industrial automation. Thus, the government’s own investment and development strategy is playing a significant role in incentivizing companies to automate, speeding the outflow of labor-intensive workers from the manufacturing industry.

In addition to manufacturing, the large influx of labor that is entering the service industry also may be flowing out of the construction industry. The Chinese state and private sector have been heavily investing in construction for decades, and from the 1990s to the 2000s, demand for construction labor rose (Garnaut et al. 2018). Large quantities of labor-intensive workers were used to build projects that range from high-speed-rail networks to expressway systems to housing projects all over the country. For example, in 2008, China began development on its first high-speed-train network, launching construction on a system that was designed to connect over 100 cities and carry over two million passengers a day (“National Trunk Highway System (NTHS)” 2020). The project was completed in 2015, with only smaller-scale extension projects currently occupying the construction industry. The extensive Chinese freeway system represents a similar situation. Recovering from a severely underdeveloped transportation system in the 1990s, China launched construction of the largest expressway system in the world (Sloboda and Yao 2007). Initially, the system was intended to be finished in 2020 but had already been completed by the mid-2000s (Mcnichol 2007). Today, nearly every major city in China is linked to the expressway network. This largely complete network means that continued investment in infrastructure is declining (Wu 2014). Finally, the housing market is experiencing a similar problem, with tens of millions of apartments built in the last decade, only to be left empty due to high costs and undesirable locations (Dong 2019; Kawase 2019). In the past several years, construction of new housing has fallen (Liu and Xiong 2018). These construction trends compound to create a construction sector with low demand for labor-intensive workers. Due to this lack of demand, construction workers are displaced into the only current viable industry for labor-intensive jobs in China, which is the informal service industry.

There are many pressures that combine to create the trends that are observed in the low-wage, labor-intensive industries. Although there is little pressure coming from agriculture, farming cannot absorb much labor in the coming years. Instead, as globalization and automation emerge as steady forces in China, economic and political issues lead to a growing stream of labor-intensive workers into the service industry from manufacturing. Likewise, falling demand for construction opens the industry as another feeder for labor-intensive workers who are flowing into the service industry, altogether a variety of contextual and economic factors that result in the exodus of labor-intensive workers from other industries into the service industry. These large flows also lead to increases in the supply of labor to labor-intensive industries that is dampening the growth of wage rates, even while demand rises.

## Emergence of Polarization in China’s Economy

Here, we summarize what the emerging employment and wage trends in the different industries and sub-economies of China in the past decade may mean for China’s future economic development. Drawing mainly from the experiences of other upper-middle-income and high-income countries, we detail the potential directions that our data suggest for China’s economic future as well as provide policy recommendations that we believe can guide China away from potential economic stagnation. The findings of our research point to the emergence of trends that, if continuing over time, will lead to the polarization of wages between China’s skill-intensive and labor-intensive labor markets.

We speculated whether the polarization trends that we have identified are new. Although previous research has focused on polarization in China, in fact, most of the papers that claimed to study polarization used data from before 2010, when labor-intensive wages were rising faster than GDP and when the coefficient was actually declining, according to some sources, e.g., Han et al. (2016). For example, in research by Wan and Wang (2015) and Meng (2012), the data series used in their analyses ended in 2007 and 2009, respectively. In essence, then, these authors were actually looking at the high levels of inequality (which is an important concern) instead of the forces that lead the dynamics of true polarization. To the best of our knowledge, the only study that truly focuses on polarization is Fleisher et al. (2018). In this paper, the authors examine the forces that were systematically pushing some groups of laborers into the informal sector while allowing others to stay in the high-wage, formal sector. Nevertheless, the analysis, which relied on simulations, and data sources (household data from 1995, 2002, and 2013) did address many of the same issues that we identified in this paper as forces that are pushing China toward an economy characterized by polarization.

We can perhaps better understand the similar sets of employment and wage-growth trends and the longer-run consequences of those trends by examining the experience of other nations and their recent economic histories. Looking first at middle-income economies, we see that China may be beginning to follow a path similar to many countries that have been caught in a middle-income trap, whereby wages polarized and growth in the overall economies either stagnated or fell. Before reaching this stage of development, many of these middle-income countries had experienced strong economic growth, due partially to their seemingly unlimited supply of low-skilled laborers (Agénor et al. 2012). When the supply of cheap labor inside their economies was eventually exhausted, and wages began to rise (i.e., trends similar to what we see in China today), a number of economic forces (e.g., globalization) began to change the organization of their economies (Kharas and Kohli 2011). Those at the lower end of the economic spectrum (e.g., workers with relatively low levels of human capital) suddenly found themselves being moved out of the (more) formal manufacturing sector, which was moving to lower-wage countries in other parts of the world (Kohli and Mukherjee 2011; Maddison 2003; Zhang et al. 2013). This created a large group of laborers who did not have the skills necessary to contribute to the emerging skill-intensive and high-wage sectors in the economies and meant that these laborers were displaced into the informal sub-economy.

In fact, these informal sub-economies, which are almost exclusively comprised of labor-intensive employment, account for large shares of national employment in these middle-income nations. For example, informal employment accounts for about 50% of employment in Argentina, 40% in Brazil, 55% in Mexico, and 35% in Chile (International Labour Organization 2013). The inequality in education between skill-intensive and labor-intensive workers contributed to economy-defining wage polarization in almost every one of these trapped-in-the-middle-income countries. Moreover, this wage polarization appears to have become a permanent feature of these economies, as, due to the low levels of human capital of those already in the informal economy (that is, adults between the ages of 20–65), there has been almost no case in which a country has been effective in retraining their workforce to allow the transformation of low-skilled workers into a profession that is employable in a skill-intensive industry (Squire 2008).

The wage polarization associated with falling into this middle-income trap has brought noticeable negative long-run effects to the economies of almost all of the countries that have fallen into the trap. In the case of Mexico, for example, due to tax and regulation avoidance in the large informal economy, the coexistence of formal and informal firms means that firms in the same industry face different marginal production costs, and this leads to an allocation of resources that undermines the rise of a formal economy. Due to the benefits to individual firms for being in the unregulated informal economy, many firms chose to exit the formal sub-economy when the benefits provided by formality were outweighed by the costs. When this occurs on an increasingly large basis, it affects the ability of the government to finance public services, leading to an accelerating undermining of the formal sub-economy (Levy 2008).

According to Enamorado et al. (2016), this deterioration of the formal economy and the weakening of the overall public infrastructure has further increased wage polarization, which has led to large increases in crime rates and further expansion of the informal economy as well as an the emergence of an illegal sub-sub-economy. Importantly, the literature indicates that such trends have not been confined to Mexico. Polarization has brought with it a generation of social tensions in many countries (including South Korea and many countries in Latin America, among others) (Cho et al. 2013; Esteban and Ray 1994; Gasparini et al. 2008).

We also consider whether China is able to overcome the middle-income trap (for example, by riding the momentum of its current fast-growing economy) and to make the transition to high-income status. Unfortunately, the problems that we describe above are not exclusive to middle-income countries but, rather, arise in high-income countries as well. Specifically, the issue of wage polarization and the problems that this has triggered have become features of economies in nations such as the USA and the UK. For example, the USA, like China, has a supply of college-educated individuals that does not meet the rising demand in the high-skilled sectors of the its economy (Carnevale and Rose 2014).

Further, globalization and automation have long been factors that have shaped decisions of firms and policymakers in the US economy, and, in recent years, these trends have become only more intense (Mason and Solís 2017). As a consequence, manufacturing has steadily declined in the USA, beginning in the 1960s. Moreover, beginning in the 1980s, the middle-skilled class in the USA started to be displaced due to automation and other forces. The demand in the high-skilled sector not only increased the need for those with ever-higher levels of human capital, which led to rising wages in the skill-intensive sector, but also the displacement of low- and middle-skill workers increased the supply of workers who had no option but to move into the service sector. This unidirectional flow of labor led to the steady deterioration of low-skilled, service-sector wages (Autor et al. 2006). Similar trends begin to be seen in many other advanced economies, such as the UK and Spain (Consoli and Sánchez-Barrioluengo 2019; Lee, Sisson and Jones 2013).

Wage polarization, even in high-income countries, brings with it many negative consequences. Initially, wage polarization has the consequence of raising inequality. In the USA, for example, from 1990 to 2018, the Gini coefficient of US household income distribution rose from 0.43 to 0.49. Similar rises were experienced in the UK. This heightened income inequality, and the forces behind the increases, in turn, have had other negative effects, such as the reductions in income mobility seen in the USA (Chetty et al. 2017). Taken together, such factors undermine expectations of a better future for many in the economy, and this has been shown to be related to a number of negative factors in high-income economies, such as a reduction in public goods, increases in psychosocial stress (Pérez and Ramos 2010), and decreases in life expectancy (Chetty et al. 2016). Hence, even if China were to reach high-income status, the trends toward economic polarization could very well continue and, as elsewhere, could have large negative economic and social consequences.

## Conclusion

After more than 10 years of rising wages, China has begun to experience divergent wage trends and large shifts in their labor-force makeup. The growth rate of skill-intensive wages has increased rapidly, while the growth rate of labor-intensive wages has begun to slow and move toward potential stagnation. Although total employment in skill-intensive industries has been increasing in recent years, informal labor-intensive industries still dominate overall employment, and employment in these industries is rising much faster than in any other sector. In addition, the distribution of labor in labor-intensive industries has undergone structural changes, as employment in manufacturing and construction in the formal economy has declined since 2013. The decline in manufacturing is due largely to globalization and automation, while housing gluts and the completion of China’s largest country-wide infrastructure projects also are responsible for the decline in the demand for construction workers. Laborers who exit the more formal manufacturing and construction industries have flowed into informal labor-intensive services, and this increase in employment, which also includes a large share of new entrants into the work force, fuels the massive expansion of the informal sector. Moreover, given the constraints to the demand for services (e.g., due to a high share of GDP going to investment, the dominance of capital-intensive industries in some sectors of the economy, and the high rates of savings of consumers due to high housing prices and low levels of social services), this rise in the supply of labor has outpaced the rise in the demand for services. These supply and demand imbalances then are behind the falling growth rates of labor-intensive wages.

Given its current trajectory of wage polarization, if China hopes to be able to escape the middle-income trap (or avoid the negative consequence of wage polarization if it can achieve high-income status), far-reaching changes need to be made to current policies, particularly in regard to education. A notable feature of high-income countries and countries that were able to escape this trap (and avoid polarization) is high base levels of education in all levels of the economy. When workers are displaced from their formal labor-intensive jobs, it is only the relatively well educated who will be able to make the transition to skill-intensive industries. In China, however, most labor-intensive workers do not have the human capital required to make this transition. Thus, the most important investment that China can make is in education, particularly in rural areas, where schooling, academic achievement, and human capital lag behind urban areas by a significant degree.

As discussed, the lack of a social safety net also contributes to the emergence of the polarization of wages that we are beginning to see in China. The lack of a comprehensive social safety net causes many middle-class consumers to save large amounts of their income rather than spend it. This constricts demand for labor-intensive services, as consumers save income that would otherwise be spent on services. If the government provided a comprehensive social safety net, the impact on the Chinese economy would be almost immediate.

For China to afford the spending that is needed to improve education and expand social services, the country may need to make some fundamental decisions in regard to where to reallocate money. One possible source may be infrastructure investment. In recent years, China has spent nearly USD 150 billion on infrastructure (Li et al. 2017). To fund rural schools and to improve social services, while expensive, China needs to shift a significant share of the infrastructure budget to these areas. Removing the Hukou restriction and allowing left-behind and rural children to be educated in cities also may contribute significantly to addressing the rural schooling problem (Wang 2017). Such investments take many years to realize, and the choices are difficult and have a degree of uncertainty. Nevertheless, the benefits of these decisions and reforms will help to bring rural schooling to the forefront of policymaking in China.

## Appendix

See Tables 9 and 10.

**Table 9:** Informal and rural–urban annual wages in China, 2008–2017. *Sources* Informal employment wage data are from the National Bureau of Statistics of China (www.stats.gov.cn). Rural–urban migrant worker data are from NBSC’s Annual Report of Monitoring Survey on National Rural–Urban Migrant Workers (NBSC, 2010–2018)

Year	Informal Employment	Rural–urban migrant employment
2008		16,080
2009	18,199	17,004
2010	20,759	20,280
2011	24,556	24,588
2012	28,752	27,480
2013	32,706	31,308
2014	36,390	34,368
2015	39,589	36,864
2016	42,833	39,300
2017	45,761	41,820

Average annual wages are in yuan

**Table 10:** Informal and rural–urban annual wage-growth rates in China 2008–2017. *Source* National Bureau of Statistics of China (www.stats.gov.cn)

Year	Informal wage growth (%)	Rural–urban migrant wage growth (%)	GDP growth (%)
2010	10.42	15.46	10.60
2011	12.23	15.03	9.60
2012	14.12	8.93	7.90
2013	10.87	11.04	7.80
2014	9.08	7.62	7.30
2015	7.29	5.78	6.90
2016	6.07	4.52	6.70
2017	5.15	4.74	6.80

Real wage-growth rates are calculated using the same data as that of Appendix Table 9 and are reduced by CPI

## Footnotes

1 Skill-intensive services include information; transmission, computer services, and software; financial intermediation; real estate; scientific research; technical service and geologic prospecting; education; health care and social welfare; and culture, sports, and entertainment.

2 Labor-intensive services include transportation and storage; wholesale and retail trades; hotel and catering services; leasing and business services; and services to households and other services.
